# Editorial: Pediatric Venous Thromboembolism

**DOI:** 10.3389/fped.2018.00269

**Published:** 2018-09-27

**Authors:** Brian R. Branchford, Julie Jaffray, Arash Mahajerin

**Affiliations:** ^1^Department of Pediatrics, Section of Hematology, Oncology at University of Colorado School of Medicine and Children's Hospital Colorado, University of Colorado Hemophilia and Thrombosis Center, Denver, CO, United States; ^2^Division of Hematology, Oncology, BMT, Department of Pediatrics, Children's Hospital Los Angeles, University of Southern California Keck School of Medicine, Los Angeles, CA, United States; ^3^Division of Hematology, Children's Hospital Orange County Children's Specialists, Orange, CA, United States

**Keywords:** pediatric, venous thromboemblism, thrombosis, child, clot

Due to the morbidity, mortality, and healthcare costs of venous thromboembolism (VTE), the focus on its treatment and prevention is growing steadily. As part of a response to the U.S. Surgeon General's 2008 call-to-action to prevent VTE (1), the Healthy People 2020 initiative included a goal to reduce the number of people who develop a VTE by at least 10% (2). The American Society of Hematology recently emphasized its focus on transforming VTE care and updated its research agenda to prioritize studies that increase understanding of VTE risk profiles based upon unique pathophysiologic mechanisms in specific clinical subgroups of patients and assess the safety and efficacy of various prevention strategies (3). Similarly, the National Heart, Lung, and Blood Institute at the National Institutes of Health includes in a recent publication of its strategic vision a focus on identifying factors that account for individual differences in VTE pathobiology and in responses to treatments (4).

The incidence of VTE in pediatric patients is increasing (rising by 70% in under a decade) (5), but the body of medical literature surrounding this topic is not keeping pace. This trend is most dramatic in hospitalized children (averaging 5–22 per 10,000 pediatric inpatients) (6–9), but community-acquired pediatric VTE is also increasing (0.1–0.5 per 10,000 children) (7, 8). The Children's Hospitals Solutions for Patient Safety collaborative recently determined that that hospital-acquired VTE is the second-largest cause of preventable harm in approximately 130 pediatric hospitals participating in this network (10, 11). VTE is associated with catastrophic short term complications, including pulmonary embolism in 15–20% (12) which confers a mortality rate of nearly 10% (13). Long term complications also occur, such as recurrent VTE or post-thrombotic syndrome (chronic pulmonary hypertension or painful limb swelling secondary to venous insufficiency) in 20% of cases (14). Finally, an episode of pediatric VTE can cost the healthcare system nearly $30,000, not only by increasing length of hospitalization by an average of 8 days, but also by creating a need for additional outpatient visits, extended drug treatment costs, and the potential for additional testing to investigate inherited or acquired thrombophilia (15–17). Drug treatment costs are likely to increase as the use of more expensive direct oral anticoagulants rises in children after ongoing dose-finding studies are completed. In response to the rising burden imparted by this disease, we designed this article collection to highlight pediatric-specific VTE risk factors, diagnosis, and treatment strategies, guidelines for risk assessment, risk-based prevention efforts, and education for the medical community about this serious, sometimes overlooked, medical condition in children.

The purpose of this Research Topic, comprising 16 articles by 35 authors, is to provide a comprehensive survey of the myriad considerations within pediatric VTE. We have welcomed worldwide thrombosis experts to write about epidemiology and risk assessment of pediatric VTE. Other specific topics include hospital-acquired VTE, community-acquired VTE, thrombosis in acutely ill children, neonates, adolescents, and those with high-risk medical issues such as cancer or congenital heart disease or specific risk factors such as central venous catheters, inherited thrombophilia, inflammation, critical illness, and trauma. Additionally, we have highlighted thrombosis in specific anatomic locations with unique diagnostic and management consideration, including abdominal veins, pulmonary embolism, and cerebral sinovenous thrombosis, as well as vascular anomalies. This Research Topic also addresses treatment for VTE in children, such as systemic and catheter-directed thrombolysis, as well as the various new oral direct-acting anticoagulants (many of which are expected to gain FDA approval for pediatrics in the coming years), for which few written guidelines exist.

Though pediatric VTE has long suffered from a paucity of high-quality evidence from which to derive practice standards and treatment/prevention guidelines, some articles in this Research Topic highlight key ongoing studies that aim to increase our knowledge in this area. One such study, mentioned in the pulmonary embolism (Zaidi et al.) and VTE treatment (Malec and Young) articles, is the Evaluation of the Duration of Therapy for Thrombosis in Children (Kids-DOTT) study that aims to determine the optimal duration of treatment (6 weeks vs. 3 months) for children with provoked deep vein thrombosis (18). Similarly, the Children's Hospital-Acquired Thrombosis registry study (19), a multi-institutional effort to retrospectively derive a pediatric VTE risk-assessment model for subsequent prospective validation, is discussed in the epidemiology/risk assessment article by Mahajerin and Croteau.

Overall, we feel this Frontiers in Pediatrics Research Topic is a unique opportunity to highlight this important topic in a single pediatric-specific resource.

## Author contributions

All authors listed have made a substantial, direct and intellectual contribution to the work, and approved it for publication.

### Conflict of interest statement

The authors declare that the research was conducted in the absence of any commercial or financial relationships that could be construed as a potential conflict of interest.
